# PREVENTion of a parastomal hernia with a prosthetic mesh in patients undergoing permanent end-colostomy; the PREVENT-trial: study protocol for a multicenter randomized controlled trial

**DOI:** 10.1186/1745-6215-13-226

**Published:** 2012-11-27

**Authors:** Henk-Thijs Brandsma, Birgitta ME Hansson, Hilde V-Haaren-de Haan, Theo J Aufenacker, Camiel Rosman, Rob P Bleichrodt

**Affiliations:** 1 Department of Surgery, Canisius Wilhelmina Hospital, Nijmegen, The Netherlands; 2 Department of Surgery, Rijnstate Hospital, Arnhem, The Netherlands

**Keywords:** Parastomal hernia, Prophylactic, Prevention, Mesh, Colostomy

## Abstract

**Background:**

Parastomal hernia is a common complication of a colostomy. Ultimately, one-third of patients with a parastomal hernia will need surgical correction due to frequent leakage or life-threatening bowel obstruction or strangulation. However, treatment remains a challenge resulting in high recurrence rates. Two single center trials demonstrated that the frequency of parastomal hernias decreases by prophylactic placement of a mesh around the stoma at the time of formation. Unfortunately, both studies were small-sized, single-center studies and with these small numbers less common complications could be missed which were the reasons to initiate a prospective randomized multicenter trial to determine if a retromuscular, preperitoneal mesh at the stoma site prevents parastomal hernia and does not cause unacceptable complications.

**Methods:**

One hundred and fifty patients undergoing open procedure, elective formation of a permanent end-colostomy will be randomized into two groups. In the intervention group an end-colostomy is created with placement of a preperitioneal, retromuscular lightweight monofilament polypropylene mesh, and compared to a group with a traditional stoma without mesh. Patients will be recruited from 14 teaching hospitals in the Netherlands during a 2-year period. Primary endpoint is the incidence of parastomal hernia. Secondary endpoints are stoma complications, cost-effectiveness, and quality of life. Follow-up will be performed at 3 weeks, 3 months and at 1, 2, and 5 years. To find a difference of 20% with a power of 90%, a total number of 134 patients must be included. All results will be reported according to the CONSORT 2010 statement.

**Discussion:**

The PREVENT-trial is a multicenter randomized controlled trial powered to determine whether prophylactic placement of a polypropylene mesh decreases the incidence of a parastomal hernia versus the traditional stoma formation without a mesh.

**Trial registration:**

The PREVENT-trial is registered at: http://www.trialregister.nl/trialreg/admin/rctview.asp?TC=2018

## Background

Colorectal cancer is, together with breast cancer, the most common malignancy in the Netherlands. The incidence of colorectal cancer was over 12,000 in the year 2009. About 28,000 patients have an enterostomy, of which roughly 60% to 70% have a colostomy (Dutch Cancer Registry, Dutch Stoma Association). About half of the patients with a colostomy develop a parastomal hernia [1,2].

Probably, the true incidence is underestimated because many of these hernias are asymptomatic. Cingi *et al*. showed that 52% of their patients with a colostomy had a parastomal hernia at clinical examination, while additional computed tomography yielded an incidence of 78% [3].

Symptoms include pain due to stretching of the abdominal wall, leakage due to poor fitting appliances, skin problems, and cosmetic complaints. Moreover, bowel obstruction and strangulation of the hernia contents may be life-threatening. Despite evolution of surgical techniques, incidence rates have not declined the past 20 years [4].

Ultimately, one-third of the patients with a parastomal hernia needs surgical correction [5,6]. Parastomal hernia repair is challenging and results vary markedly between techniques. Suture repair, narrowing the opening in the fascia, is considered an obsolete procedure because the recurrence rates are over 70%. Relocation of the stoma is associated with a recurrence rate of 33% with an additional risk of developing an incisional hernia in the midline or at the old ostomy site of 20% [2,7-9]. Nowadays, prosthetic repair is the gold standard of parastomal hernia repair. Several techniques have been developed having similar results with respect to morbidity and recurrence rate (Hansson *et al.*, 2012 [10]). In the last decade, laparoscopic repair of PSH is developing. Basically two techniques are used, the modified Sugarbaker technique and the keyhole technique, of which the last seems to have a significantly higher risk of recurrence.

Because of the high incidence, inconsistent results of available data on parastomal repair and lack of sufficient treatment options, surgeons started focusing on prevention of the hernia with local reinforcement of the abdominal wall using a prosthetic mesh. At time of writing the PREVENT-trial protocol in 2009, only a few reports on this topic were published.

Two recent reviews showed that parastomal hernias can be prevented by the placement of a preperitoneal, retromuscular mesh around the stoma [11,12]. Randomized trials from Jänes and Serra-Aracil, both using a light-weight polypropylene mesh in a preperitoneal retromuscular position, found significantly more parastomal hernias in the group with a conventional stoma (53.7%) as compared to the mesh group (14.8%; *P* <0.001). Mesh related complications are rare. Serra reported one patient with a peristomal infection and one with a stenosis of the stoma. Jänes reported no mesh-related complications.

The percentage of patients with a parastomal hernia who required surgical intervention decreased in the mesh group in comparison with the non-mesh group. Both studies combined seven out of 29 patients who developed a PSH in the non-mesh group required surgical repair *versus* none of the eight in the mesh group with a PSH [13,14] (Additional file 1: Table S1).

Unfortunately the trials were small, 27 patients per group. Although a meta-analysis offers compensation for this flaw, sample size still be too small for detecting a difference when events occur infrequent. With these small numbers of patients less common complications could be missed. Furthermore the risk of bias increases due to a variability of clinical factors and non-uniform reporting of clinical parameters such as stoma site, patient characteristics, and type of surgery all contributed to the heterogeneity. To make more reliable statements on the actual decline of the incidence of PSHs, larger groups are needed.

Due to these shortcomings there is need for more methodologically sound trials.

## Methods

### Study objectives

The aim of this single-blind, multicenter, randomized controlled trial is to determine if parastomal herniation is prevented by the prophylactic placement during open surgery of a polypropylene mesh around a colostomy. Patients are blinded and are not aware if mesh placement did occur. Most surgeons will see their own patients postoperatively in the outpatient clinic so double blinding was not feasible and could not be guaranteed. Patients are randomized into two groups. In one group a preperitoneal, retromuscular positioned polypropylene mesh is placed around the stoma. In the control group a conventional stoma is created.

It is hypothesized that mesh placement will reduce the incidence of parastomal hernia of 30% down to 10%.

#### Primary endpoint

The primary endpoint is the incidence of parastomal hernia, either symptomatic or asymptomatic. In the final report, these two groups, symptomatic and asymptomatic will also be reviewed separately to see if there are more asymptomatic hernia in the mesh group.

#### Secondary endpoints

Secondary endpoints are perioperative morbidity, including stomal necrosis, stenosis, parastomal, or laparotomy wound infections. Mortality, pain, quality of life, and cost-effectiveness are other secondary endpoints that will be analyzed.

### Patient sample size

Based on available literature it is hypothesized that 30% of patients will develop a parastomal hernia, the majority in the first few years. Based on published data it is assumed that parastomal hernia will occur in 10% in the study group receiving the prophylactic mesh.

This study is powered to reveal significant differences between the two study groups. With an Alpha error of 5% (two-sided) and a Beta error of 0.10 (power of 90%), 67 patients need to be included in each arm of the trial. We decided to include a total of 150 patients which are randomly allocated in both groups. Analysis of recorded Prismant data estimate that 600 colostomies are constructed each year in the Netherlands. It is expected that the inclusion period will take 30 months and 14 hospitals are required to participate. The study started in the spring of 2010.

### Setting

Patients receiving a permanent end-colostomy in an elective setting will be recruited from the following centers:

Canisius Wilhelmina Hospital, Nijmegen; Radboud University Nijmegen Medical Centre, Nijmegen; Rijnstate Hospital, Arnhem; Maxima Medisch Centrum, Veldhoven; St Antonius Hospital, Nieuwegein; Catharina Hospital, Eindhoven; AMC Amsterdam, Amsterdam; OLVG, Amsterdam; University Medical Center Utrecht; Utrecht; Isala Clinics, Zwolle; Erasmus Medical Center, Rotterdam; Slingeland Hospital, Doetinchem; Medisch Spectrum Twente, Enschede; Albert Schweitzer Hospital, Dordrecht, The Netherlands.

The total trial period is estimated to be 7 years; the recruitment period will be 2 years, followed by a 5-year follow-up period.

### Inclusion criteria

• Patients undergoing formation of a permanent end-colostomy in an elective setting regardless of benign or malignant disease.

• Age between 18 and 85 years.

• Signed informed consent.

• Able to understand the study questionnaires.

### Exclusion criteria

• Expected survival <12 months.

• Stoma formation in an emergency setting.

• Formation of an ileostomy.

• Correction of a previous constructed colostomy.

• Previous surgery at the colostomy site.

### Ethical considerations

This trial is conducted in accordance with the Declaration of Helsinki and ‘Good Clinical Practice Guidelines’. It is approved by the Medical Ethics Committee of Nijmegen (CMO-ABR 22695). All local Medical Ethics Committees approved the final protocol.

Patients willing to participate in the trial will be provided with a patient information sheet and a reconsideration period. They will be included after written informed consent is obtained.

The PREVENT-trial is registered at:

http://www.trialregister.nl/trialreg/admin/rctview.asp?TC=2018

### Reporting

All results will be reported according to the CONSORT 2010 statement.

### Randomization

Randomization will be performed by telephone using an interactive voice response system. Patients are randomized by computer, treatment will be stratified and blocked by center to ensure each center has similar numbers of patients allocated to one of the two treatment groups.

### Safety and quality control

The trial coordinator will monitor all centers in order to identify non-compliance to protocol and serious adverse events (SAEs). SAEs are defined as any event leading to major complications and/or prolonged hospital stay due to the placement of the mesh. SAEs will be reported to the Data Safety Monitoring Board and to the accredited Medical Ethical Board (METC).

A Data Safety Monitoring Board will perform interim safety analyses and make recommendations regarding the conduct of the study to the accredited METC and the trial committee. When the mesh related complication rate is higher than 15%, the trial will be terminated.

### Preoperative work-up

In an outpatient setting all participants receive information and guidance by a stomal therapy nurse. Stoma site marking is performed by a stomal therapy nurse prior to surgery. Colonic lavage will be performed if necessary. In both groups preoperative antibiotic prophylaxis will be given according to the local agreements.

### Surgical techniques

A light-weight monofilament polypropylene mesh, Parietene Light™ (Covidien®) is the mesh of choice for this study. This mesh is chosen because there is level 2b evidence that shrinkage of the mesh, postoperative foreign body sensation, and pain are less in comparison with traditional polypropylene mesh [15,16].

**Figure 1:**
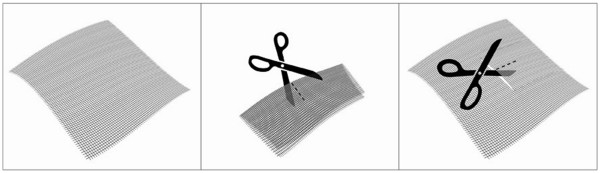
Preparing the central hole for the bowel to pass through, no central portion is cut away.

According to the technique of Jänes and Israëlsson, the intended bowel for the colostomy is closed with a stapling device, thus minimizing the chance of contamination. The trephine is created by excision of the skin-oval at the preoperatively marked ostomy site. No subcutaneous tissue is excised. After exposing the anterior rectus sheath, a cross-shaped incision is made in the fascia. The rectus abdominus muscle is split in the direction of the fibers. In the mesh group a retromuscular space is created and dissected to the lateral border via the median laparotomy. The posterior fascia/peritoneum is left undisturbed. A 10×10 cm Parietene Light™ mesh, with a cross-shaped incision in the center of the prosthesis to allow passage of the colon loop, is placed on the posterior rectus sheath (Figure 1).

**Figure 2:**
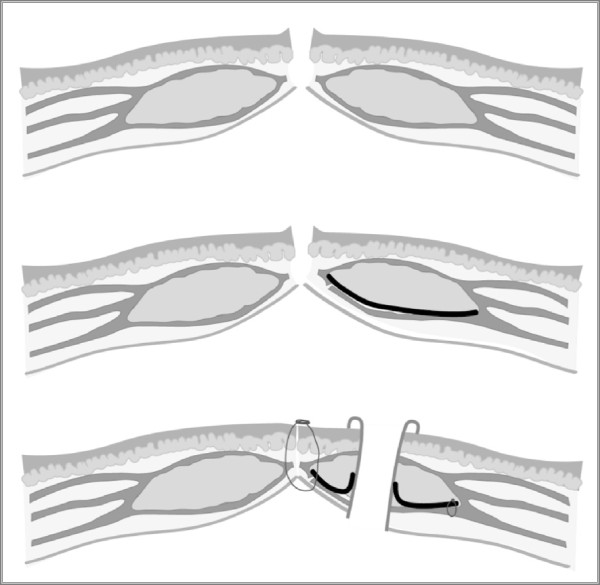
**Cross-section through the abdominal wall.** The mesh is placed in the retromuscular plane on the posterior rectus sheath/peritoneum. The bowel is passed through the pre-shaped hole in the mesh and the rectus muscle. With the closure of the abdominal wall the mesh and the peritoneum are included in the running suture.

The lateral corners of the mesh are fixed with two absorbable monofilament sutures. Then the posterior fascia is opened over the trephine in the mesh and the bowel is gradually passed through. Closing the midline incision, the running suture includes the medial border of the mesh and the peritoneum, thus preventing contact between the mesh and the viscera (Figure 2). The stoma has to protrude 1 to 2 cm above the skin and is fixed with resorbable sutures to the skin [13].

This technique is described in detail in the study protocol to ensure standardized mesh placement. It is available in text and on video on our website: http://www.preventtrial.nl.

### Follow-up and definitions of complications

Outpatient follow-up is scheduled at 3 weeks, 3 months, 1, 2 and 5 years postoperatively. Postoperative complications, such as peristomal infections, degree of stomal ingrowth, and leakage are recorded.

Parastomal hernia is defined as any detectable bulge in the vicinity of the ostomy with the patient erect, supine, and performing the Valsalva maneuver. Prolapse is scored if significant prolabation of bowel occurred causing the stoma to increase in length without peri-stomal bulging. Wound infection is defined in deep, superficial, or peristomal infections using the C.D.C criteria for surgical site infection [17].

Stomal dehiscence is defined as separation of the bowel mucosa from the skin, measured in millimeters. Stomal necrosis is defined as ischemia of the mucosal tissue. Stenosis is defined as narrowing of the stomal trephine leading to obstruction. Leakage is present if stomal material has to be replaced more than once every 2 days.

If there is a clinical or physical suspicion of a hernia a CT scan will be performed in a supine position with use of the Valsalva maneuver.

Quality of life and postoperative pain is determined using validated health scores preoperatively and during all moments of follow-up after the index operation.

### Health status scores

The SF-36 is a validated multi-purpose, short-form health survey. It yields an eight-scale profile of functional health and wellbeing scores as well as physical and mental health summary measures and a preference-based health utility index [18,19]. Completed preoperatively, 1 year, and 5 years after index operation.

Questionnaire of von Korff for Grading the Severity of Chronic Pain [20]. Three months and 1 year after the index operation.

EuroQoL-5D is an instrument which calculates an index which gives a societal-based quantification of the patients health status combined with a visual analogue scale [21]. This so-called health-related quality of life (HRQoL) instrument will be completed during every moment of follow-up. This index gives a societal-based global quantification of the patient’s health status.

### Cost analysis

The cost analysis exists of two main parts. First, on patient level, volumes of care will be measured prospectively using case record forms. Per arm (intervention and control) full cost-prices will be determined using activity based costing. Productivity losses for patients (sick leave) will be estimated by using the case record forms (CRFs). The friction cost-method will be applied following the Dutch guidelines [22].

The second part of the cost analysis consists of determining the cost prices for each volume of consumption in order to use these for multiplying the volumes registered for each participating patient. The Dutch guidelines for cost analyses will be used. For units of care/resources where no guideline or standard prices are available real cost prices will be determined.

### Data collection

All data will be collected in personal CRFs, which will be stored in the patient’s own hospital. Copies of the completed forms will be sent to our coordinating center (Canisius-Wilhelmina Hospital, Nijmegen). All data will be stored in a double-entry database (SAS). Independent monitoring visits will be performed throughout the entire duration of the trial. When patients are not treated according to their allocation, for any reason, they will stay in the trial following the Intention-to-Treat-principle.

### Trial status

The PREVENT-trial is currently open for recruitment. We expect to reach our powered number of included patients in the coming months.

## Abbreviations

CRF: Case record form; PSH: Parastomal hernia; RCT: Randomized Controlled trial.

## Competing interests

The authors declare that they have no competing interests.

## Authors’ contributions

HB and BH were involved in trial coordination and drafted the manuscript. All authors contributed to the design and development of the trial protocol. All authors approved the study and this manuscript.

## Supplementary Material

Additional file 1: Table S1 Data from all randomised controlled trials regarding prevention of parastomal hernia’s (PSH) with a peristomal retromuscular mesh [13,14]. Click here for file
