# Supplementary material for: PREVENTion of a parastomal hernia with a prosthetic mesh in patients undergoing permanent end-colostomy; the PREVENT-trial: study protocol for a multicenter randomized controlled trial
Source: Trials. 2012 Nov 27;13:226. doi: 10.1186/1745-6215-13-226 (PMC3576295; doi:10.1186/1745-6215-13-226)
Supplement: Additional file 1: Table S1 — Data from all randomised controlled trials regarding prevention of parastomal hernia’s (PSH) with a peristomal retromuscular mesh [13,14]. [file 1745-6215-13-226-S1.pdf]

Table S1: data from all randomised controlled trials regarding prevention of parastomal hernia's (PSH) with a peristomal retromuscular mesh.

	**Follow-up (months)**	**Repair**	***n***	**PSH *n* (%)**	**Complications *n* (%)**
Jänes *et al.* 2009 [11]	65 (57-83)	Polypropylene mesh (Vypro®)	27	2 (12%)	None
		Non-mesh	27	17 (81%)	None
Serra-Aracil *et al.* [12]	29 (13-49)	Polypropylene mesh (Ultrapro®)	27	6 (22%)	Peristomal infection 1 (3.7 %) Stoma necrosis 1(3.7%)
		Non-mesh	27	12 (45%)	Peristomal infection 1 (3.7 %) Stoma necrosis 1 (3.7%)
